# Near-infrared multispectral photoacoustic microscopy using a graded-index fiber amplifier

**DOI:** 10.1016/j.pacs.2016.08.002

**Published:** 2016-08-16

**Authors:** Takashi Buma, Jessica L. Farland, Margaret R. Ferrari

**Affiliations:** a Department of Electrical and Computer Engineering, Union College, Schenectady, NY 12308, USA; b Bioengineering Program, Union College, Schenectady, NY 12308, USA

**Keywords:** Photoacoustic microscopy, Stimulated Raman scattering, Graded-index multimode fiber

## Abstract

We demonstrate optical resolution photoacoustic microscopy (OR-PAM) of lipid-rich tissue using a multi-wavelength pulsed laser based on nonlinear fiber optics. 1047 nm laser pulses are converted to 1098, 1153, 1215, and 1270 nm pulses via stimulated Raman scattering in a graded-index multimode fiber. Multispectral PAM of a lipid phantom is demonstrated with our low-cost and simple technique.

## 1 Introduction

Photoacoustic microscopy (PAM) [1], [2] of lipid-rich tissue, such as atherosclerotic plaques [3], [4], [5], [6] or myelinated peripheral nerves [7], [8], typically requires near-infrared laser wavelengths near 1210 nm or 1720 nm. These wavelengths correspond to the second and first overtone optical absorption of C—H bonds in lipids [7]. Unfortunately, neither of these wavelengths are produced by commonly available pulsed lasers (e.g. Nd:YAG). Therefore, expensive optical parametric oscillators (OPOs) are necessary to perform PAM of lipids. OPOs have high pulse energy, large wavelength tuning range, and repetition rates in the several kHz range. However, the very high cost and slow wavelength tuning are major drawbacks for practical applications, where multiple wavelengths are preferred to distinguish lipids from surrounding tissue.

We have been exploring techniques based on nonlinear fiber optics to develop cost-effective lasers for PAM of lipid-rich tissue. Nanosecond laser pulses from a fixed-wavelength laser are sent through an optical fiber, where nonlinear propagation produces multiple wavelengths. The wavelength distribution depends on the laser pulse parameters and fiber properties. When nanosecond pulses pump an optical fiber near the zero-dispersion wavelength, extreme spectral broadening occurs due to several nonlinear mechanisms such as modulation instability, soliton dynamics, and phase-matched four-wave mixing [9]. Photonic crystal fibers pumped in this manner produce a supercontinuum spanning from visible to near-infrared wavelengths. In contrast, an optical fiber pumped in the normal dispersion region produces discrete wavelengths via stimulated Raman scattering (SRS) [10].

We demonstrated the first application of both techniques for PAM, where the desired wavelength is selected by a band-pass filter [11], [12]. Supercontinuum generation has very broad wavelength coverage but low pulse energy (e.g. 150 nJ or less) in a specific spectral band (e.g. 10 nm width) [11], [13], [14]. SRS concentrates energy into fewer discrete wavelengths, leading to higher pulse energy [15], [16]. Hajizera performed in-vivo blood oxygenation PAM at 532, 545, 558, and 590 nm using a high repetition rate fiber laser with an optical fiber [16]. We recently demonstrated this technique for near-infrared PAM, where a birefringent optical fiber converted a 1064 nm laser to 1097, 1150, 1215, 1275, and 1325 nm [17].

A major advantage of our technique is its simplicity, as it uses a low-cost pulsed laser followed by an optical fiber. However, an important drawback is the low pulse energy, typically a few hundred nJ [17]. Such low pulse energies require significant signal averaging for optical-resolution PAM (OR-PAM) of lipids. In this paper, we demonstrate an improved system with an over 10-fold increase in laser pulse energy at 1215 nm, corresponding to the optical absorption peak of lipids. Pulse energies of several μJ make it possible to image lipid-rich tissue with little to no signal averaging, an important step towards practical applications.

## 2 Methods

### 2.1 Near-infrared multi-wavelength pulsed laser

**Fig. 1:**
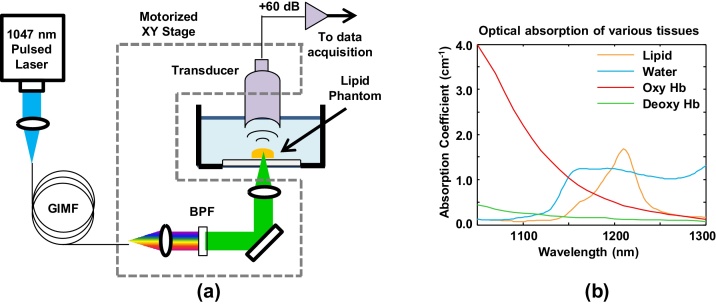
(a) Schematic of the multi-wavelength pulsed laser based on a graded-index multimode fiber (GIMF). A dielectric band-pass filter (BPF) selects the desired wavelength for OR-PAM. The components within the dashed box are mounted on a motorized 2-D positioning stage. (b) Near-infrared optical absorption spectra of lipid (orange), water (blue), oxy-hemoglobin (red), and deoxy-hemoglobin (green) [18], [19].

Fig. 1a shows the schematic of our multi-wavelength pulsed laser. A Q-switched Nd:YLF laser (CrystaLas Corp) produces 140 mJ pulses at a wavelength of 1047 nm. The pulse duration is 14 ns and the repetition rate is 1 kHz. The laser pulses are coupled into 95 m of a graded-index multimode fiber (GIMF) with a 50 μm core diameter and 0.22 numerical aperture (GIF-50C, Thorlabs). The fiber output is collimated with a 15 mm focal length aspheric lens and sent through a filter wheel containing hard-coated band pass filters (Edmund Optics) at 1050, 1100, 1150, 1225, and 1275 nm. The 1050 nm filter has a 10 nm bandwidth and 80% transmission. All other filters have a 50 nm bandwidth and 80% transmission.

Although pulsed Nd:YLF lasers at 1053 nm are more common, systems operating at 1047 nm are also commercially available. For our particular system, the 1047 nm wavelength is important to generate pulses at 1215 nm, which lies close to the optical absorption peak of many lipids (Fig. 1b) [18], [19]. Our technique relies on stimulated Raman scattering (SRS), which describes the interaction of a laser with the vibrational modes of an optical medium. SRS produces a series of frequency down-converted “Stokes lines” separated by 13.2 THz, corresponding to the peak of the Raman gain spectrum in fused silica [10], [20]. An input laser at 1047 nm propagating through a silica optical fiber should produce Stokes lines at 1098, 1153, 1215, and 1284 nm.

The GIMF core has a parabolic refractive index, in contrast to the uniform profile of conventional step-index optical fibers [21]. Despite being a multimode fiber, a GIMF has several advantages. First, it exhibits an interesting “beam clean-up” property, where an input pump laser with poor spatial beam quality produces Stokes wavelengths with good spatial beam quality [22], [23]. This is clearly an important feature for achieving fine spatial resolution in OR-PAM. Second, the larger diameter core permits scaling to higher pulse energies by suppressing optical damage. Furthermore, higher energy Stokes pulses can develop before saturation occurs [17]. Third, robust and high fiber coupling efficiency (e.g. 65%) is easily achieved without elaborate positioning mounts.

### 2.2 OR-PAM system

Fig. 1a also shows our transmission-mode OR-PAM system, where the focused optical excitation and acoustic detection are on opposite sides of the sample. The selected wavelength from the multi-wavelength source is focused with a 50 mm focal length achromat. The photoacoustic signal is detected by a 25 MHz f/2 transducer (Olympus NDT), amplified by 60 dB (Miteq), sent through a 50 MHz low pass filter (Mini-Circuits), and finally acquired by a digitizer board operating at 250 MS/s (National Instruments). Two-dimensional scanning of the object is performed with a computer controlled positioning system (Velmex). Data acquisition is performed with LabVIEW while data processing and reconstruction are performed off-line in MATLAB.

## 3 Experiments and results

### 3.1 Spectral characterization of the GIMF output

**Fig. 2:**
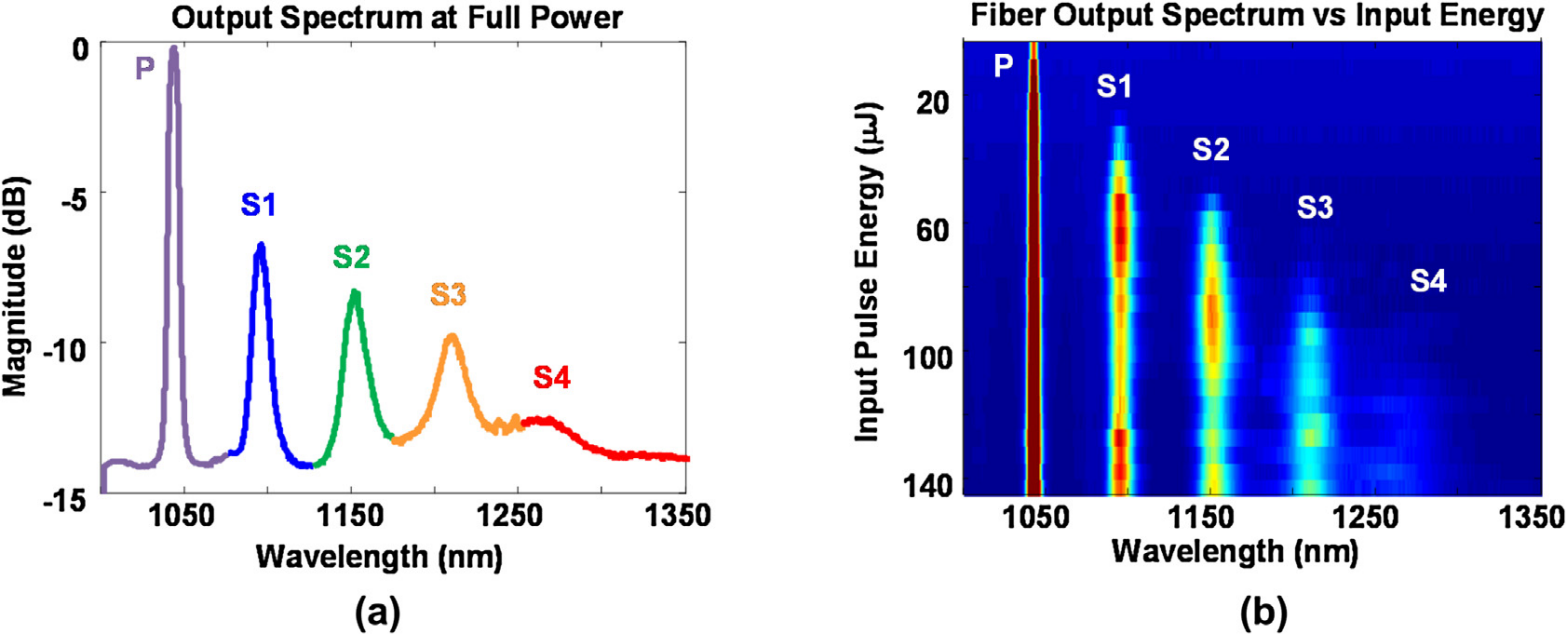
(a) Measured spectrum of the optical fiber output at full power, corresponding to an input pulse energy of 140 μJ. The third Stokes line S3 at 1215 nm lies close to the optical absorption peak of lipids. (b) Measured fiber output spectra with input pulse energy varied from 10 to 140 μJ. A new Stokes line is produced for every 30 μJ increase in input pulse energy.

The spectral properties of the GIMF output were measured with a scanning monochromator (Optometrics). Fig. 2a shows the spectrum at “full power”, corresponding to an input pulse energy of 140 μJ. The input pump (P = 1047 nm) and first three Stokes lines (S1 = 1098 nm, S2 = 1153 nm, S3 = 1215 nm) are clearly visible. The measured Stokes wavelengths closely agree with the expected values from SRS in fused silica. The Stokes line S4 is lower in peak spectral intensity but much broader in bandwidth. The expected peak wavelength of S4 is 1284 nm, but the actual value is near 1270 nm. This broadening and peak shift are most likely due to the presence of phase-matched four-wave mixing (FWM). FWM describes the nonlinear interaction between two input waves at frequencies f_1_ and f_2_ to produce two new waves at frequencies f_3_ and f_4_. Efficient FWM occurs near 1300 nm in silica fibers, where low chromatic dispersion permits phase matching between all four waves [20].

Approximately two-thirds of the laser energy coupled into the fiber remains at 1047 nm, while the remaining third is distributed among the Stokes wavelengths. Table 1 shows the measured pulse energy after each dielectric band-pass filter. The pulse energy is 7 μJ after the 1225 nm filter (50 nm bandwidth), corresponding to the Stokes peak at 1215 nm (S3). This energy is over ten times higher than our previous system [17]. Although peak S3 has a fairly broad linewidth (32 nm), we have successfully demonstrated multispectral PAM of lipids with this laser source. The spectral broadening of the higher Stokes orders is most likely due to FWM [10], [20].

In theory, SRS produces a new Stokes line when the pump power is increased by a discrete amount [20]. This threshold behavior is confirmed in Fig. 2b, where the fiber output spectrum is shown at various input laser energy levels. A new Stokes line is produced when the incident pulse energy is increased by approximately 30 μJ. The actual SRS threshold energy inside the fiber is estimated to be 6.5 μJ, considering the 65% fiber-coupling efficiency and our previous observation that only one-third of the fiber-coupled pump contributes to SRS.

A rough theoretical estimate of the threshold energy can be computed in the following manner. SRS exhibits a “critical” intensity given by I_cr_ ≈ 16/(g_R_L_eff_), where g_R_ is the Raman gain coefficient and L_eff_ is the effective fiber length [20]. The corresponding pulse energy can then be estimated by E_cr_ = I_cr_ A_eff_ τ_P_, where A_eff_ is the effective mode area inside the fiber and τ_P_ is the laser pulse duration. Assuming g_R_ = 0.92 × 10^−11^ cm/W [24] and L_eff_ = 95 m, the critical intensity is I_cr_ = 183 MW/cm^2^. The GIMF fundamental mode-field diameter is estimated to be 13 μm, based on a Gaussian approximation [25]. Using A_eff_ = π (6.5 μm)^2^ and assuming a pulse duration of τ_P_ = 14 ns, the theoretical critical energy is E_cr_ ≈ 3.4 μJ. A_eff_ is probably larger due to the pump laser occupying a few fiber modes, so it is not surprising that this theoretical estimate is lower than the experimental value (6.5 μJ).

### 3.2 Spatial characterization of the GIMF output

**Fig. 3:**
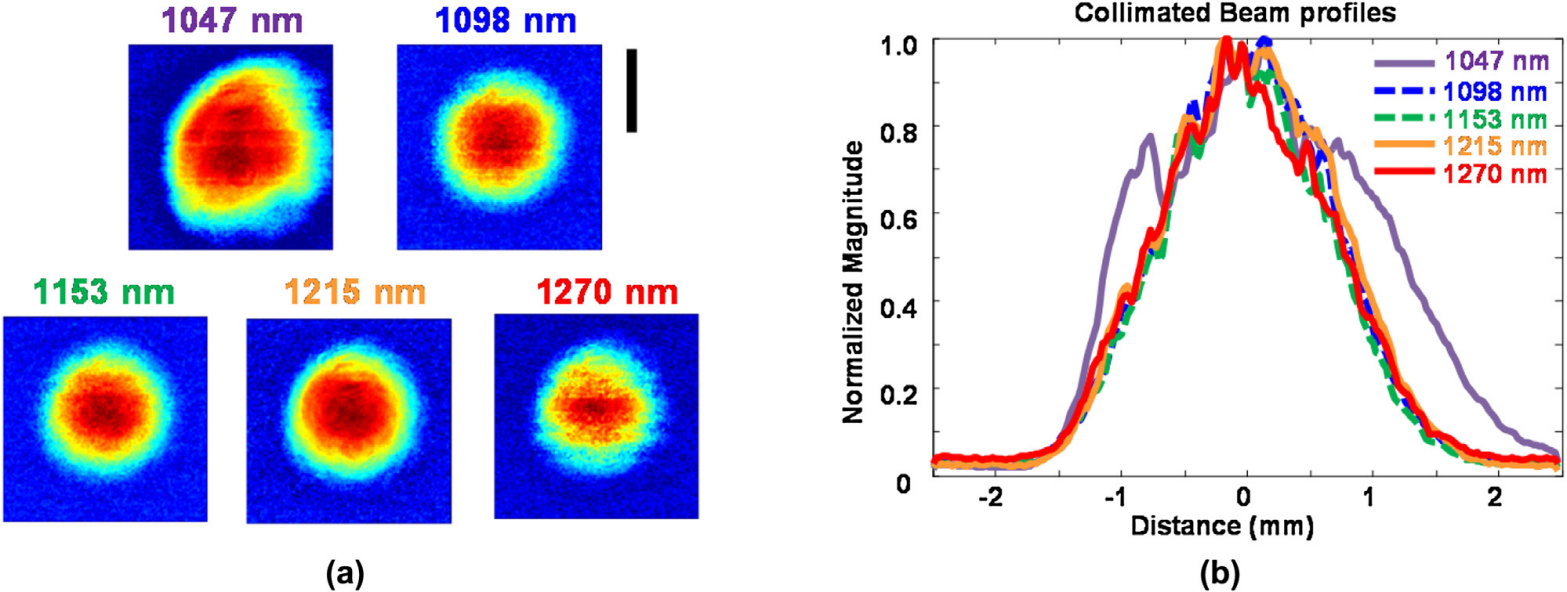
(a) Spatial beam patterns of the collimated GIMF output for each wavelength. Each image covers a 5 × 5 mm region. The scale bar represents 2 mm. (b) Beam profiles obtained from horizontal slices through the center of each beam pattern.

In order to confirm the “beam clean-up” properties of the GIMF, the spatial beam quality of the collimated fiber output was measured by raster scanning the tip of a multimode fiber (50 μm core) connected to an InGaAs photodiode. Fig. 3a shows the measured collimated beam patterns over a 5 × 5 mm region. The pump wavelength (1047 nm) does not have a smooth Gaussian shape. In contrast, the Stokes lines have a tighter and more circular pattern. Fig. 3b shows horizontal slices of the beam patterns, confirming that the Stokes lines have very similar profiles while the pump beam is broader. The Stokes lines fit very well to a Gaussian profile, suggesting a fundamental TEM_00_ (transverse electromagnetic) spatial mode. The same analysis along the vertical direction produces similar results.

It is possible for a laser beam with a Gaussian-like profile to not be purely TEM_00_ but instead contain many spatial modes. A more accurate assessment of beam quality is the M^2^ parameter, often called the “beam propagation parameter” [26]. A TEM_00_ beam has M^2^ = 1 and produces the minimum possible optical focus. A beam with a mixture of spatial modes has M^2^ > 1 and produces a larger optical focus. We measured M^2^ by analyzing the focused laser beam width at 20 positions along the propagation direction, in compliance with the ISO 11146 standard [27]. Due to the lack of an infrared CCD or CMOS camera, we measured beam profiles by raster scanning the tip of a single mode fiber connected to an InGaAs photodetector.

**Fig. 4:**
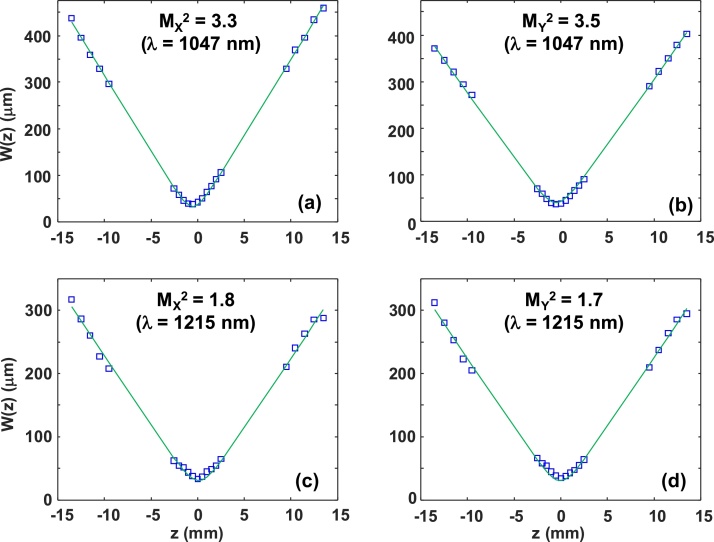
Measurements of the beam propagation parameter of the GIMF output at two wavelengths. The evolution of the beam width during propagation is analyzed along the x-axis and y-axis separately. (a) M_X_^2^ and (b) M_Y_^2^ at 1047 nm. (c) M_X_^2^ and (d) M_Y_^2^ at 1215 nm. The measured data points are shown in blue squares, while the fitted curves are shown in solid green.

Fig. 4 shows the M^2^ measurements of the GIMF output at two different wavelengths. The original laser wavelength at 1047 nm clearly has larger M^2^ values (M_X_^2^ = 3.3 and M_Y_^2^ = 3.5) than the third Stokes line at 1215 nm (M_X_^2^ = 1.8 and M_Y_^2^ = 1.7). This is consistent with the results from Fig. 3 and confirms the beam clean-up property of SRS. Both M_X_^2^ and M_Y_^2^ are greater than 1 at λ = 1215 nm, suggesting that SRS in a GIMF does not produce a pure TEM_00_ mode. Considering that the GIMF supports hundreds of guided modes, it is not surprising that beam clean-up results in the “survival” of several modes localized at the center of the fiber core. Nevertheless, M^2^ < 2 is still a reasonable value for beam quality and is consistent with other studies [28], [29]. We did not measure M^2^ at other wavelengths, due to the time-consuming nature of the measurement procedure. However, the similar beam profiles of all the Stokes wavelengths in Fig. 3 suggest that their respective M^2^ values should be similar as well. This assumption is validated in our OR-PAM spatial resolution measurements described in subsection 3.3.

### 3.3 OR-PAM spatial resolution

**Fig. 5:**
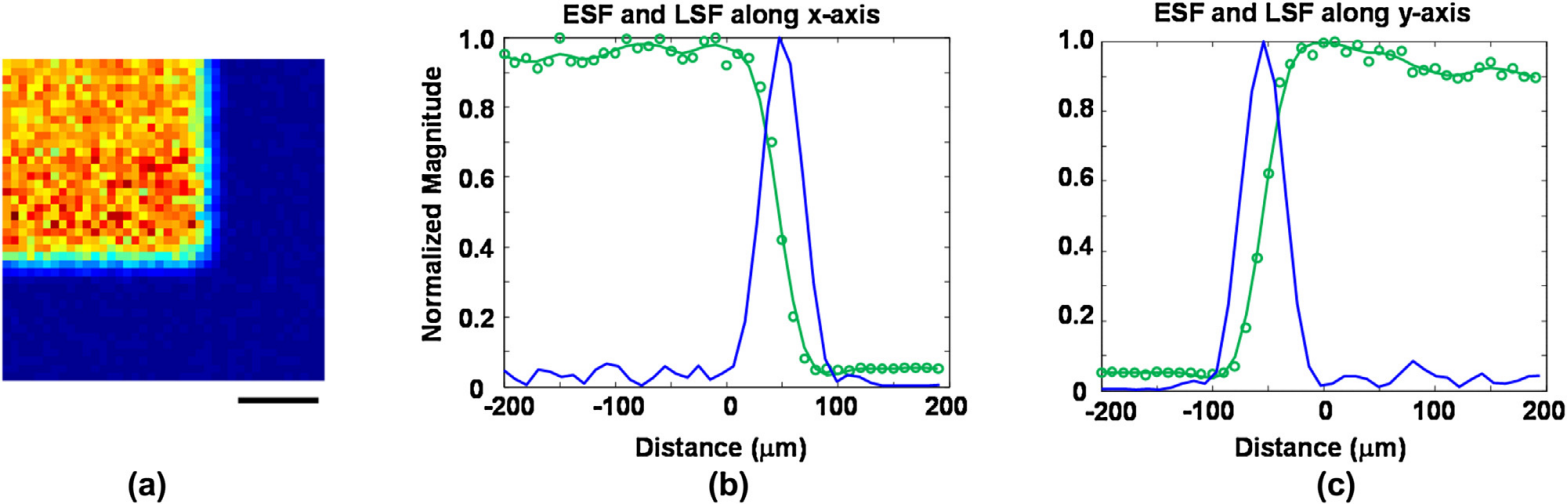
(a) OR-PAM image (λ = 1215 nm) of a corner of the Group 2, Element 1 pattern of a USAF target immersed in 1 mm of water. The image covers a 400 × 400 μm region, and the scale bar represents 100 μm. (b) The edge spread function (ESF) and line spread function (LSF) along the horizontal dimension. (c) ESF and LSF along the vertical direction.

The lateral resolution of our OR-PAM system was measured by imaging the corner of the Group 2, Element 1 pattern of a United States Air Force (USAF) target through 1 mm of water. A 40 × 40 pixel scan using a 10 μm step size was recorded for each filter wavelength. Signals were averaged 100 times to improve the accuracy of measuring the edge spread function (ESF). The pulse energy was reduced by a factor of 20 to prevent optical damage to the target. The C-mode image at 1215 nm is shown in Fig. 5a. An ESF was obtained by averaging ten rows and curve fitting with a smoothing spline in MATLAB. The derivative of the fitted curve produced the line spread function (LSF). Lateral resolution was defined as the full width at half-maximum (FWHM) of the LSF.

Table 2 shows the measured spatial resolution for each wavelength. As expected, the spatial resolution is worst at 1047 nm and approximately the same at all other wavelengths. “Beam clean-up” is a cumulative process, so it is not surprising that subsequent Stokes lines have cleaner spatial beams and therefore slightly smaller values of spatial resolution. If the laser is assumed to propagate purely in the GIMF fundamental mode, we estimate the minimum theoretical resolution to be 28 μm at λ = 1215 nm. Accounting for M^2^ = 1.7 increases the theoretical estimate to 48 μm, in reasonable agreement with our OR-PAM measurement (44 μm).

Axial resolution was characterized by measuring the FWHM of the envelope of the photoacoustic signal from the USAF target. Time-to-depth conversion was achieved by measuring the shift in signal arrival time due to a 50 μm displacement of the target depth. The axial resolution was measured to be 66 μm, which is consistent with the approximately 50% fractional bandwidth (pulse-echo) of the 25 MHz receive transducer. The measured axial resolution was the same for all wavelengths. This is consistent with our findings that the laser pulse duration at the GIMF output is roughly the same (14 ns) at all wavelengths. Higher Stokes orders had slightly shorter pulse duration (e.g. 1 ns shorter) than the input pump laser. This is most likely due to the intensity dependent nature of SRS.

### 3.4 Multispectral OR-PAM of lipid phantom

**Fig. 6:**
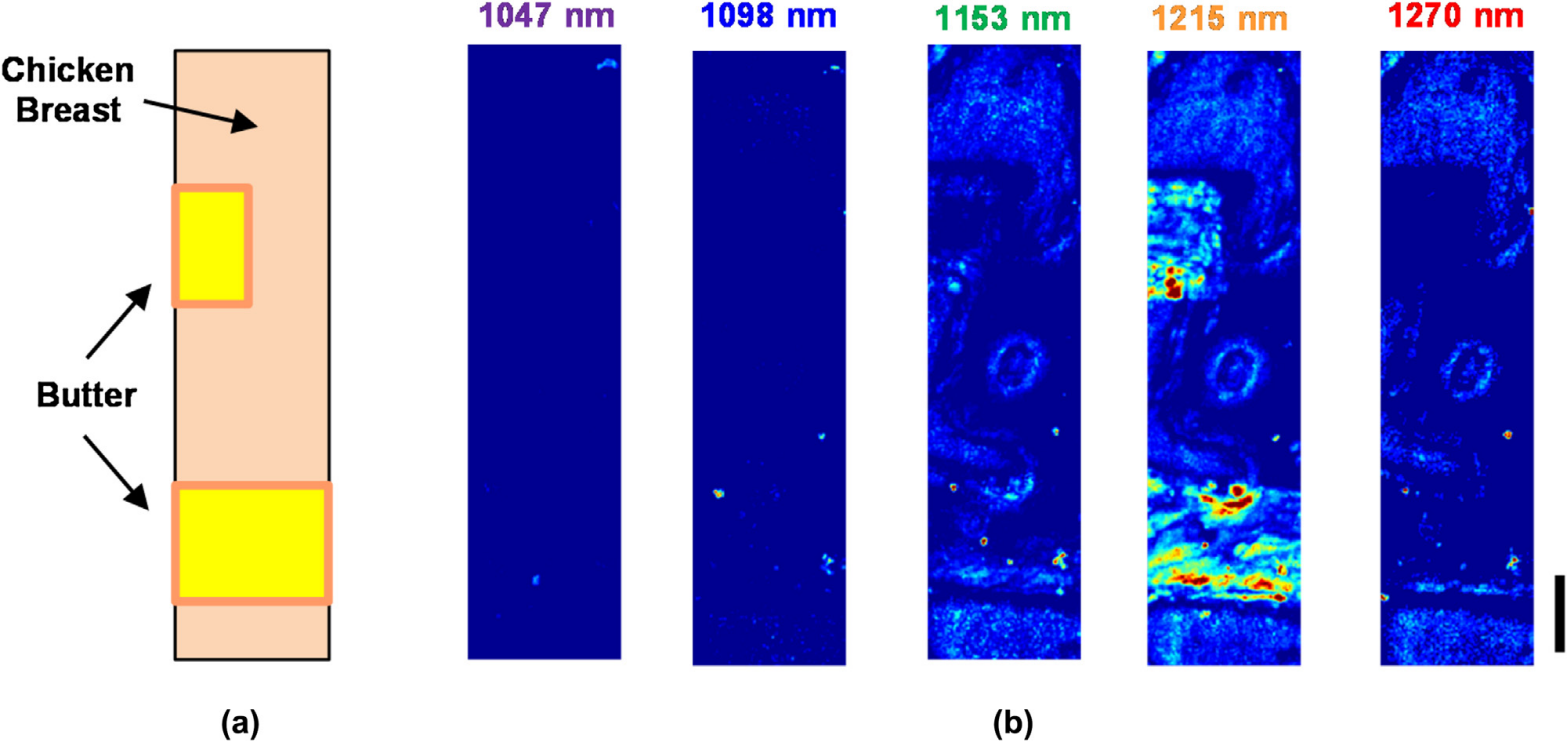
(a) Schematic of the lipid phantom used for multispectral OR-PAM. (b) Maximum amplitude projection images at 1047, 1098, 1153, 1215, and 1270 nm. The scale bar represents 500 μm.

Multispectral OR-PAM was performed on two thin strips (thickness ≈ 0.5 mm) of butter sandwiched between approximately 0.8 mm thick layers of chicken breast meat. The phantom was imaged with a 400 × 100 pixel scan using a 10 μm step size and no signal averaging. Fig. 6 shows the maximum amplitude projection images at 1047, 1098, 1153, 1215, and 1270 nm. Each image shows the received photoacoustic signal amplitude, scaled according to the ratio of pulse energy at each wavelength (Table 1) with respect to 1215 nm. All images are then displayed over the same linear range as the 1215 nm image. Images were not compensated for laser energy fluctuations during data acquisition. The butter strips are clearly most prominent at 1215 nm and still visible at 1153 nm. The surrounding tissue has a different wavelength dependence, where they appear roughly the same brightness at 1153, 1215, and 1270 nm.

**Fig. 7:**
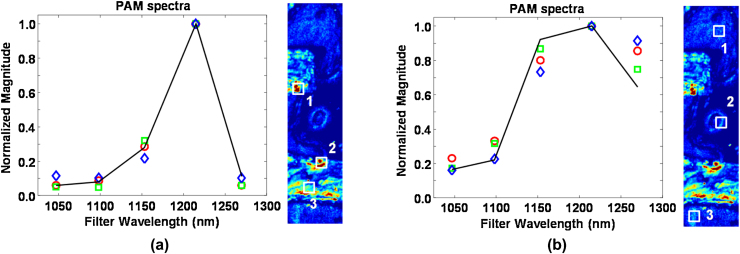
(a) Photoacoustic spectra taken at three different locations within the two lipid objects. Locations 1, 2, and 3 correspond to the red circles, green squares, and blue diamonds, respectively. The black curve is the reference spectrum based on the lipid absorption coefficient μ_lipid_[18] and the measured laser spectrum. (b) Photoacoustic spectra at three different locations surrounding the lipid objects. The black curve is the reference spectrum based on μ_water_[18] and the measured laser spectrum.

A more quantitative comparison of the wavelength dependence between the two regions is shown in Fig. 7. Fig. 7a shows photoacoustic spectra at three different locations within the butter regions of the phantom. At each location, the spectra are averaged over a 2 × 2 pixel region to reduce variations from pulse-to-pulse fluctuations of the laser. All plots are normalized with respect to the 1215 nm component. The spectral profiles are consistent between all three locations. For validation purposes, the photoacoustic spectra are compared to a reference spectrum computed by multiplying the lipid absorption coefficient μ_lipid_ [18] with the measured laser spectrum and integrating within the pass band of each dielectric filter. Fig. 7a shows reasonable agreement between the photoacoustic and reference spectra.

A similar analysis was performed on three regions outside of the butter, as shown in Fig. 7b. As expected, the photoacoustic spectra are clearly different from butter and are most likely due to optical absorption from water. This hypothesis is supported by the solid line in Fig. 7b, showing the theoretical photoacoustic spectrum from water. The greater variability at 1270 nm is most likely caused by the degraded image SNR due to the low laser pulse energy.

The sensitivity of our OR-PAM system was characterized by computing the SNR of each photoacoustic signal recorded from the three butter regions identified in Fig. 7a. The single-shot SNR from all 4 pixels within each region were then averaged together. The averaged single-shot SNR from regions 1, 2, and 3 is 21, 23, and 20 dB, respectively. This is a major improvement compared to our previous system, where the low laser pulse energy required averaging over 100 signals in order to obtain similar SNR values [17]. The laser fluence at the optical focus inside our lipid phantom is estimated to be 400 mJ/cm^2^. A four-fold reduction would be required to comply with the ANSI limit of surface fluence at near-infrared wavelengths (100 mJ/cm^2^) [30]. However, the resulting SNR loss can be offset by using a higher NA detection transducer.

## 4 Discussion

Our GIMF-based multi-wavelength laser produces pulse energies of several μJ, making it highly suitable for high resolution intravascular photoacoustic (IVPA) systems employing optical focusing [6]. The reasonable beam quality (M^2^ ≈ 1.7) of our laser allows finer lateral resolution (e.g. below 10 μm) with stronger focusing. Despite the relatively broad linewidth (>30 nm) of our laser pulses, our results suggest that in vivo multispectral PAM of lipids should be feasible.

Fast imaging requires data acquisition with little to no signal averaging, so fluctuations in laser pulse energy can affect image quality. The laser energy fluctuation over 10,000 pulses was measured to be 2% and 23% for the 1047 nm input and the 1215 nm output, respectively. This increase in variability is not surprising, since the nonlinear nature of SRS tends to exacerbate input laser fluctuations. Pulse-to-pulse fluctuations directly translate to pixel intensity variations in the resulting image. This can be mitigated by using a photodiode to monitor the GIMF output, and would be a straightforward improvement to our current system.

Further improvements to our system for practical multispectral PAM applications include higher repetition rate, flexible wavelength tuning, narrow spectral linewidth, and rapid wavelength tuning. The repetition rate of our current system is limited by our 1 kHz pump laser. The large GIMF core can easily accommodate 10 kHz systems, but scaling to 100 kHz lasers will require a more careful study of laser-induced damage to the fiber.

**Fig. 8:**
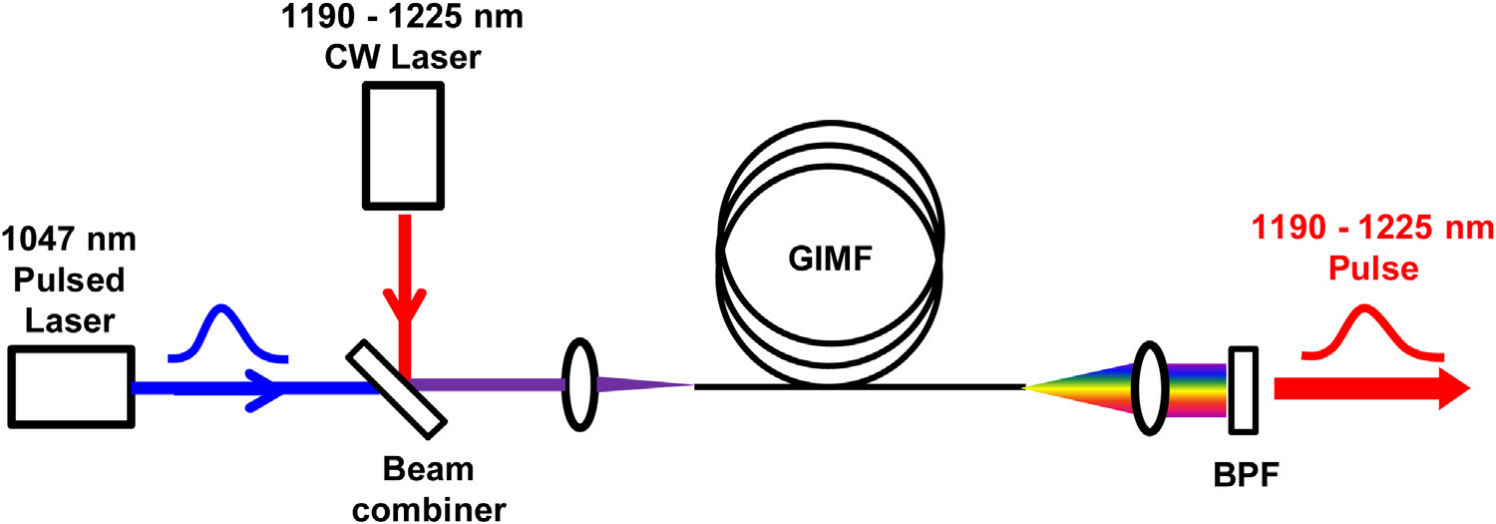
Schematic of a possible tunable optical source to perform PAM between 1190 and 1225 nm. Both the pulsed 1047 nm Nd:YLF laser and a CW tunable laser (1190–1225 nm) are coupled into the GIMF. The second Stokes line (S2 = 1153 nm) amplifies the CW laser via SRS. The band-pass filter (BPF) selects the resulting laser pulses between 1190–1225 nm.

Our current system produces a fixed set of wavelengths. Continuous wavelength tuning between 1190 and 1225 nm could be possible by combining two lasers (“pump” and “signal”) inside the fiber, as shown in Fig. 8. The “pump” consists of 1047 nm pulses producing Stokes lines at 1098 nm (S1) and 1153 nm (S2) in the usual manner. The “signal” is a continuous-wave (CW) laser that is tunable between 1190 and 1225 nm. The pulsed S2 amplifies the weak signal via SRS to produce a high power pulsed output at the signal wavelength. Amplification over a wide wavelength range is possible due to the broad bandwidth of the Raman gain in fused silica [10]. Only modest signal power (e.g. 30 mW) is needed for amplification by SRS [31], making semiconductor lasers an attractive implementation. Although the linewidth of the pulsed output (e.g. <1 nm) would be somewhat broader than the CW signal input [31], it is still a significant improvement compared to our current system.

The slow wavelength selection of our current system is limited by the dielectric filter wheel. Millisecond-scale wavelength switching could be achieved with an acousto-optic tunable filter (AOTF). One potential pitfall to this approach is a large optical insertion loss, since the Stokes linewidths (20–30 nm) of our current system are considerably wider than the AOTF spectral resolution (e.g. 5 nm). This drawback could be mitigated by the narrow linewidth pulses produced by the injection-seeded amplifier technique depicted in Fig. 8.

Our technique is scalable to higher energies. The key is to use an optical fiber that has both: (1) single-mode (or nearly single-mode) propagation for efficient SRS (2) a large mode area for large saturation energy. The GIMF in our experiments does emulate single-mode behavior for SRS, but the mode field diameter (≈13 μm) is not dramatically higher than conventional single-mode fibers. Yb-doped fibers with mode field diameters exceeding 80 μm have been used to produce high energy amplification of nanosecond pulses [32]. This would be sufficient to produce SRS pulse energies of a few hundred μJ. SRS techniques using bulk optics, such as barium nitrite [33], may be more suitable for producing mJ-level pulses near 1200 nm for deep-tissue acoustic-resolution PAM [2].

## 5 Conclusions

We have demonstrated a pulsed multi-wavelength laser source based on stimulated Raman scattering in a graded-index multimode fiber (GIMF). The μJ-level pulses at 1215 nm have sufficient beam quality for in-vivo OR-PAM of lipid-rich tissue. Our technique is attractive for practical applications due to the simple apparatus and scalability to higher repetition rates. Future work will concentrate on extending our technique to develop a more narrowband and continuously tunable pulsed laser to possibly identify specific lipids using multispectral OR-PAM.

## Conflict of interest

None.

## Funding source

National Science Foundation grant.

## Figures and Tables

**Table 1:** Pulse energy and linewidth vs Wavelength.

Spectral Peak	P (1047 nm)	S1 (1098 nm)	S2 (1153 nm)	S3 (1215 nm)	S4 (1270 nm)
Filter center wavelength (nm)	1050	1100	1150	1225	1275
Filter bandwidth (nm)	10	50	50	50	50
Pulse Energy (μJ)	60	12	9	7	3
−3dB Linewidth (nm)	4	10	17	32	–

**Table 2:** Spatial resolution vs Wavelength.

Spectral Peak	P (1047 nm)	S1 (1098 nm)	S2 (1153 nm)	S3 (1215 nm)	S4 (1270 nm)
Resolution (X) (μm)	63	54	46	43	43
Resolution (Y) (μm)	62	50	49	44	47
